# Novel instructionless eye tracking tasks identify emotion recognition deficits in frontotemporal dementia

**DOI:** 10.1186/s13195-021-00775-x

**Published:** 2021-02-08

**Authors:** Lucy L. Russell, Caroline V. Greaves, Rhian S. Convery, Jennifer Nicholas, Jason D. Warren, Diego Kaski, Jonathan D. Rohrer

**Affiliations:** 1 Dementia Research Centre, Department of Neurodegenerative Disease, UCL Queen Square Institute of Neurology, London, WC1N 3BG UK; 2 Department of Medical Statistics, London School of Hygiene and Tropical Medicine, London, UK; 3 Centre for Vestibular and Behavioural Neurosciences, Department of Clinical and Motor Neurosciences, UCL Queen Square Institute of Neurology, London, UK

**Keywords:** Behavioural variant frontotemporal dementia, Emotion recognition, Eye tracking, Social cognition, Ventromedial prefrontal cortex, Orbitofrontal cortex

## Abstract

**Background:**

Current tasks measuring social cognition are usually ‘pen and paper’ tasks, have ceiling effects and include complicated test instructions that may be difficult to understand for those with cognitive impairment. We therefore aimed to develop a set of simple, instructionless, quantitative, tasks of emotion recognition using the methodology of eye tracking, with the subsequent aim of assessing their utility in individuals with behavioural variant frontotemporal dementia (bvFTD).

**Methods:**

Using the Eyelink 1000 Plus eye tracker, 18 bvFTD and 22 controls completed tasks of simple and complex emotion recognition that involved viewing four images (one target face (simple) or pair of eyes (complex) and the others non-target) followed by a target emotion word and lastly the original four images alongside the emotion word. A dwell time change score was then calculated as the main outcome measure by subtracting the percentage dwell time for the target image before the emotion word appeared away from the percentage dwell time for the target image after the emotion word appeared. All participants also underwent a standard cognitive battery and volumetric T1-weighted magnetic resonance imaging.

**Results:**

Analysis using a mixed effects model showed that the average (standard deviation) mean dwell time change score in the target interest area was 35 (27)% for the control group compared with only 4 (18)% for the bvFTD group (*p* < 0.05) for the simple emotion recognition task, and 15 (26)% for the control group compared with only 2 (18)% for the bvFTD group (*p* < 0.05) for the complex emotion recognition task. Worse performance in the bvFTD group correlated with atrophy in the right ventromedial prefrontal and orbitofrontal cortices, brain regions previously implicated in social cognition.

**Conclusions:**

In summary, eye tracking is a viable tool for assessing social cognition in individuals with bvFTD, being well-tolerated and able to overcome some of the problems associated with standard psychometric tasks.

**Supplementary Information:**

The online version contains supplementary material available at 10.1186/s13195-021-00775-x.

## Introduction

Behavioural variant frontotemporal dementia (bvFTD) is a neurodegenerative disorder characterised by a progressive decline in behaviour and executive function [1, 2]. One of the key early features is an impairment in social cognition, a set of skills that underlies our interactions with others [3], and includes emotion recognition, the ability to identify the emotions of others, e.g. from their facial expression.

Emotions are often split into simple or basic ones, which are universally recognised cross-culturally and include happiness, sadness, fear, disgust, anger and surprise, and complex ones such as regret and distrust. Individuals with bvFTD have been found to recognise emotions less accurately than healthy controls [4, 5], especially those of negative valence such as anger, sadness, fear and disgust [6–8], as well as more complex ones [9–13]. However, traditional emotion recognition tasks are often ‘pen and paper’ and use complex instructions with high working memory load that may be difficult for those with cognitive impairment to understand. We therefore aimed to develop novel tasks of emotion recognition using the methodology of eye tracking [14–16]. This has previously been used to investigate oculomotor function in FTD [17–19], and more recently, cognition as well [20, 21]. Importantly, it can provide a quantitative output and, potentially, a more sensitive way to detect impairment within a cognitive domain than traditional tasks can. Furthermore, it can remove much of the cognitive demand of the tasks by limiting the instructions required [22].

This study therefore set out to, firstly, develop simple and complex emotion recognition instructionless eye tracking tasks which have the potential to quantitatively detect earlier and more subtle social cognition deficits than previous tests and then, secondly, explore the utility of these novel tasks in individuals with bvFTD relative to a healthy control group, as well as determining their cognitive and neuroanatomical correlates.

## Methods

### Participants

Forty participants were recruited from the longitudinal FTD studies at the Dementia Research Centre, University College London: 18 people meeting diagnostic criteria for bvFTD [2] of whom 9 had genetic FTD (mutations in *C9orf72* = 5, *GRN* = 2 and *MAPT* = 2), and 22 healthy controls. The groups were of similar age, but compared to controls, a greater proportion of the bvFTD group were male and the educational level was slightly higher in the controls compared with the bvFTD group (Table 1).

**Table 1:** Demographic, behavioural and neuropsychometric data for the control and bvFTD participants. Behavioural symptoms are scored as 0 (absent), 0.5 (very mild or questionable), 1 (mild), 2 (moderate) and 3 (severe), with mean (standard deviation) scores shown for the bvFTD group. Significant differences between groups are highlighted in bold. *SD* standard deviation, *N/A* not applicable, *s* seconds

	Controls	bvFTD
	Mean (SD)	Mean (SD)
Number of participants	22	18
Sex (% male)	59%	72%
Age (years)	64.2 (5.7)	63.9 (5.7)
Education (years)	**16.8 (2.3)**	**13.4 (3.1)**
Behavioural symptoms		
Apathy	N/A	1.7 (0.7)
Disinhibition	N/A	1.7 (0.8)
Loss of empathy	N/A	1.9 (0.8)
Change in appetite	N/A	1.8 (0.9)
Obsessive-compulsive behaviour	N/A	1.8 (0.7)
CDR with NACC FTLD sum of boxes	**0.8 (0.8)**	**10.3 (3.7)**
MMSE (/30)	**29.5 (0.7)**	**24.8 (4.1)**
WMS-R Digit span forwards (/12)	**9.0 (2.2)**	**7.0 (2.3)**
WMS-R Digit span backwards (/12)	**8.3 (2.6)**	**4.8 (2.0)**
Phonemic fluency (1 min)	**15.1 (5.7)**	**8.6 (4.8)**
D-KEFS Color-Word Interference Test (ink colour time, s)	**56.5 (17.3)**	**93.3 (36.4)**
Trail Making Test part A (time, s)	**30.3 (11.2)**	**51.7 (29.9)**
Trail Making Test part B (time, s)	**69.2 (24.7)**	**171.5 (90.9)**
Graded Naming Test (/30)	**25.9 (2.9)**	**13.8 (8.9)**
Mini-Social and Emotional Assessment total (/30)	**25.6 (1.6)**	**20.1 (3.1)**
Mini-Social and Emotional Assessment Faux-Pas subtest (/15)	**12.9 (1.2)**	**10.2 (2.1)**
Mini-Social and Emotional Assessment Emotion Recognition subtest (/15)	**12.7 (1.4)**	**9.8 (1.9)**

All participants underwent a clinical and cognitive assessment including the Clinical Dementia Rating Scale with the National Alzheimer’s Coordinating Centre Frontotemporal Lobar Degeneration component (CDR with NACC FTLD), the Mini-Mental State Examination (MMSE), WMS-R Digit span forwards and backwards, Phonemic fluency, D-KEFS Color-Word Interference Test (part 3), Trail Making Test parts A and B, Graded Naming Test and the Mini-Social and Emotional Assessment (mini-SEA, which includes two subtests, a Faux-Pas task and an Emotion Recognition task). The bvFTD group performed significantly worse on all tests than the control group (Table 1).

### Eye tracking tasks

All eye tracking tasks were performed on the Eyelink 1000 Plus (SR research) with the participant’s chin on a head mount to ensure stability within a dark room to keep consistent lighting conditions. The 18″ display screen had a resolution of 1920 × 1080 pixels and was positioned 70 cm from the participant. Viewing was binocular but only the right eye was tracked. A 9-point calibration was carried out prior to the start of the tasks, followed by a drift correct procedure between each trial in order to maintain accuracy of the eye tracker throughout the task. If the accuracy was poor, recalibration was performed.

Initially, a pro-saccade task (with 8 trials) was performed to assess basic oculomotor function and therefore participant’s ability to perform the emotion recognition tasks [19, 23]. A red cross was shown in the middle of the screen, and then once the participant had fixated on the cross, there was a gap of 200 ms followed by the appearance of a green dot at either 8° visual angle in the horizontal direction or 5° visual angle in the vertical direction either side of the target fixation cross. Participants were asked to look as quickly and as accurately as they could to the green dot when it appeared. Saccade latency, the time taken for an individual to generate the initial saccade after the target has appeared; amplitude error, i.e. how close to the target an initial saccade amplitude is; and peak velocity, the maximum velocity reached for the saccade, were all calculated.

**Fig. 1:**
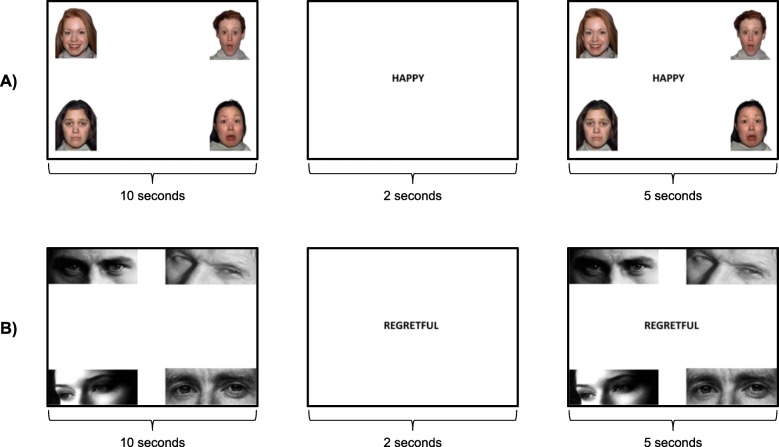
Examples of the stimuli for the **a** simple emotion recognition task and **b** complex emotion recognition task

Two tasks were developed to assess simple and complex emotion recognition. For both of these emotion recognition tasks, participants were presented with a fixation cross. Once they had looked at this, four images exhibiting particular emotions (faces for the simple task and eyes for the complex task) appeared in each of the corners of the screen for 10 s. A target emotion word then appeared in the centre of the screen for 1 s. This emotion word matched one of the four previous images. Lastly, the original four images reappeared on the screen for 5 s alongside the target word (Fig. 1). Display timings were guided by the visual world paradigm literature [24]. Participants were told only to look at the images on the screen with no other instructions. In total, the test took 10–15 min to complete.

For the simple emotion recognition task, the images used were selected from the NimStim Face Stimuli Set (https://www.macbrain.org/resources.htm) and included faces displaying the six basic emotions of happiness, surprise, sadness, disgust, anger and fear. There was a total of 24 trials with each of the emotions being the target image on four occasions (Fig. 1a). For the complex emotion recognition task, images were selected from the Reading the Mind in the Eyes Task [25], a test containing pictures of eyes with associated complex emotion labels such as contemplative and suspicious (Fig. 1b). There was a total of 20 trials for this task.

To analyse the data after the tasks had been performed, each of the four images was selected as an *interest area* and the participant’s *dwell time* within each interest area (i.e. how long they had spent looking at that image) was measured both before and after the emotion word was presented on the screen. As the length of image presentation was different before (10 s) and after (5 s) the emotion word was presented, a *percentage dwell time* was calculated as:

$$ \mathrm{Dwell}\ \mathrm{time}\ \left(\%\right)=\frac{\mathrm{dwell}\ \mathrm{time}}{\mathrm{presentation}\ \mathrm{time}}\times 100 $$

Performance on each trial was measured by the difference between percentage dwell time in the interest area of the image showing the target emotion *after* presentation of the emotion word compared to *before* it was presented. This measure was calculated as:

$$ \mathrm{dwell}\ \mathrm{time}\ \mathrm{change}\ \mathrm{score}=\mathrm{dwell}\ \mathrm{time}\ \left(\%\right)\ \mathrm{post}-\mathrm{dwell}\ \mathrm{time}\ \left(\%\right)\ \mathrm{pre} $$

The hypothesis was that controls would look approximately equally at all four images before the emotion word appeared but then spend more time looking at the target image and less time at the other three images after the emotion word appeared, i.e. a positive dwell time change score for the target, whereas people with an impairment of emotion recognition would look more equally at all four images after the emotion word appeared (as well as before), i.e. the dwell time change score would be near to zero.

A dwell time change score was also calculated for the other three images. These images were chosen to consist of one ‘similar’ image of the same valence as the target (i.e. a positive emotion if the target was positive, or a negative emotion if the target was negative) and two ‘distractor’ images of the opposite valence to the target (i.e. two negative emotions if the target was positive, and vice versa). The two distractor change scores were averaged together to give one total distractor dwell time change score. The hypothesis was that controls would have a negative dwell time change score for these non-target interest areas whereas people with emotion recognition problems would again have a score close to zero.

For each participant, the dwell time change scores were averaged across all of the trials, giving a *mean dwell time change score* as a summary measure of performance for target, similar and distractor images within each task.

For each group, we then calculated the mean of the participants’ mean dwell time change scores. To avoid double use of the word mean, and therefore for easier readability, we use the word average here, i.e. the overall group result is the *average mean dwell time change score*.

### Structural brain imaging

All participants underwent volumetric T1-weighted imaging in a Siemens Prisma 3T magnetic resonance imaging scanner. An automated atlas segmentation propagation and label fusion strategy known as Geodesic Information Flow [26] was used to parcellate the T1-weighted scans from each participant to generate specific regions of interest (ROI): orbitofrontal cortex; dorsolateral prefrontal cortex (DLPFC); ventromedial prefrontal cortex (VMPFC); temporal, parietal and occipital cortices; striatum and amygdala. All of the ROI volumes are expressed as a percentage of total intracranial volume, computed with SPM12 (Statistical Parametric Mapping, Welcome Trust Centre for Neuroimaging, London, UK) running under Matlab R20014b (Mathworks, USA) [27].

### Statistical analysis

All eye tracking data was loaded into the Eyelink 1000 Plus Data Viewer (SR Research) for pre-processing and then exported to Stata (version 14.2) for statistical analysis. Normality was assessed using Q-Q plots.

For the pro-saccade task, a saccade report was generated, and the first saccade that met the following criteria was used for the analysis: the first saccade that did not contain a blink, did not start before the onset of the target, went in the same direction as the target and started at the fixation cross. Linear regression models were used to compare saccade latency, amplitude error and peak velocity between groups (bootstrapping with 1000 replications was used for the latter two measures as they were not normally distributed).

For both the simple and complex emotion recognition tasks, a mixed effects model was used to compare the mean dwell time change scores between the two groups for each of the types of interest area (i.e. target, similar or distractor). The model therefore included participant group, type of interest area and their interaction, with the dwell time change score for the interest areas on each trial as the outcome variable. Age, sex and education were included as covariates in the analysis. Crossed random effects for participant and trial number (i.e. 1–24 for the simple emotion recognition task and 1–20 for the complex emotion recognition task) were included to allow for correlations between repeated measures on the same participant and correlations between responses to the same trial by different participants. As the data were not normally distributed, bootstrapping with 1000 replications, clustered on participant, was used to provide non-parametric bias-corrected accelerated confidence intervals for statistical inference.

For the simple emotion recognition task only, similar mixed effects models with bootstrapping were performed to investigate whether the mean dwell time change score for the target interest area differed both between and within groups for each of the different emotions.

To investigate the cognitive and neuroanatomical correlates of the simple and complex emotion recognition tasks, a correlation analysis with inference based on bootstrap standard errors from 1000 replications was performed in the bvFTD group between the dwell time change score for the target interest area and (i) the neuropsychological tests (including cognitive domains that potentially may correlate with the eye tracking tasks, i.e. social cognition, executive function, speed of processing and language) and (ii) the MRI ROI volumes (including specific neuroanatomical regions that have previously been implicated as being part of a social cognition network, with the inclusion therefore of specific frontal subregions).

## Results

No differences were observed between the bvFTD group and the controls in any of the measures on the pro-saccade task (Supplementary Table 1).

**Fig. 2:**
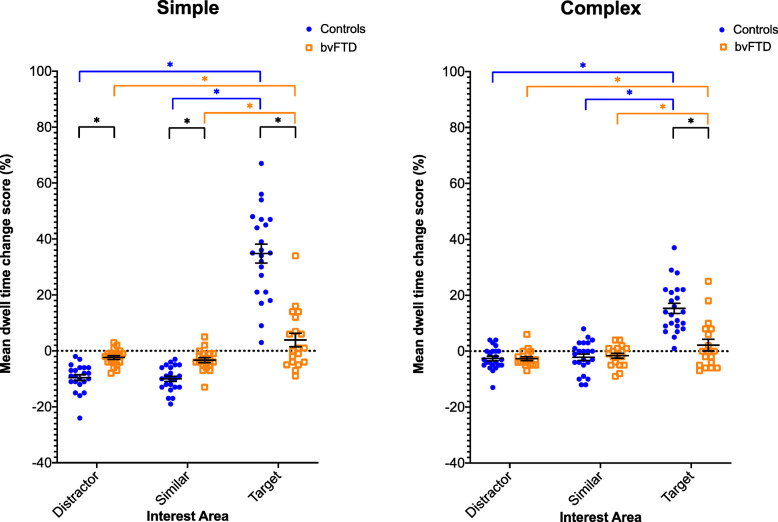
Mean dwell time change scores for bvFTD and control groups in the simple and complex emotion recognition tasks. Black significance lines indicate *between*-group differences, whilst orange and blue significance lines indicate *within*-group differences (bvFTD and controls respectively)

**Fig. 3:**
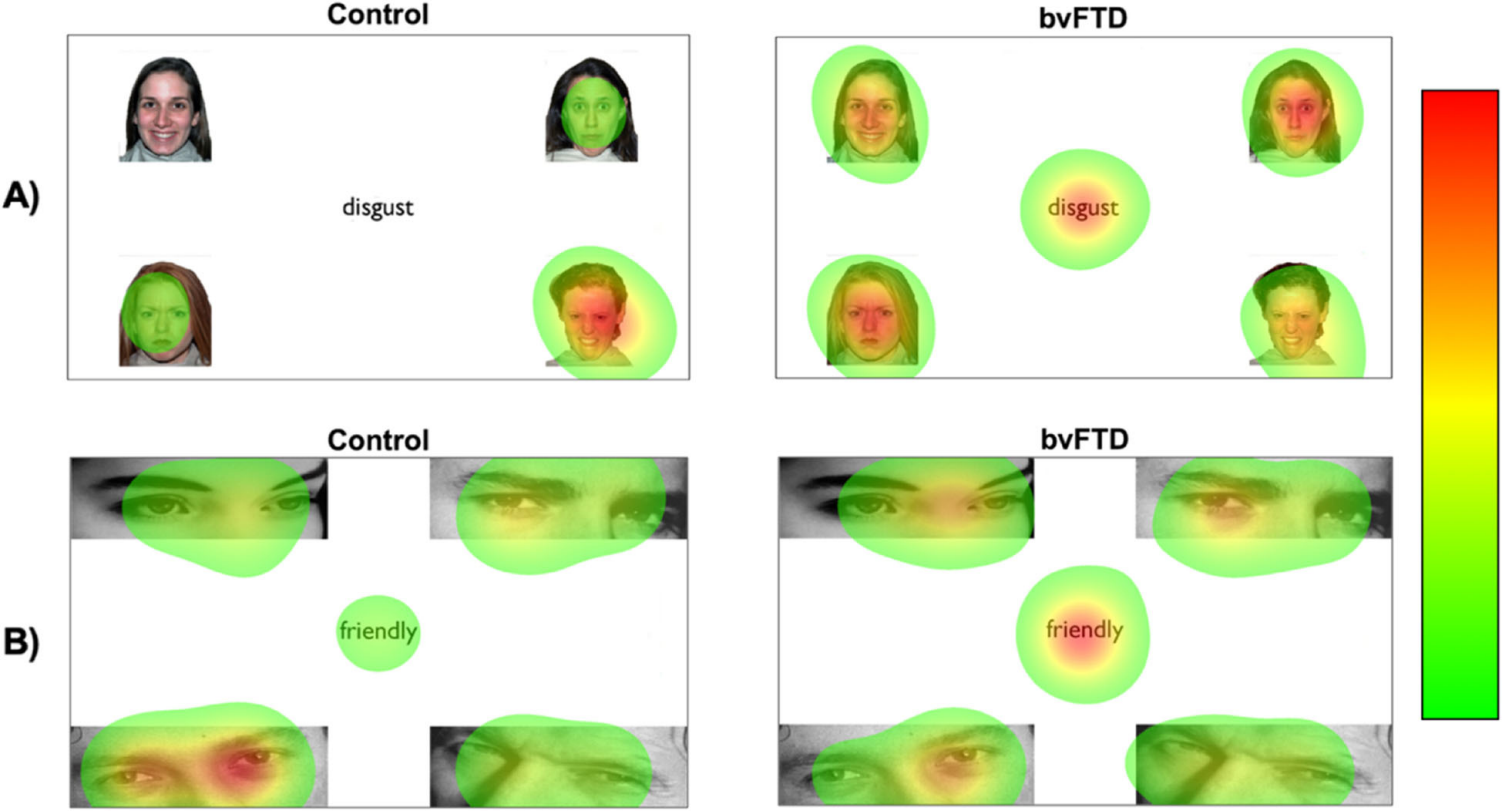
Heat maps showing average performance of controls and bvFTD participants on example trials from the **a** simple emotion recognition task and **b** complex emotion recognition task. The colour bar shows the time spent looking at a particular area in milliseconds after the emotion word is presented, where red is the most time spent. The controls look significantly more at the target image after the emotion word is presented, whereas the bvFTD participants look to a lesser extent at the target image

In the simple emotion recognition task, the control group spent significantly more time looking at the target image after the emotion word was presented than the bvFTD group (*p* < 0.05): the average (standard deviation) mean dwell time change score in the target interest area was 35 (27)% for the control group compared with only 4 (18)% for the bvFTD group (Table 2, Figs. 2 and 3). The control group also spent significantly less time looking at the similar and distractor images after the emotion word was presented than the bvFTD group: the average (standard deviation) mean dwell time change score in the similar interest area was − 10 (15)% for the control group compared with − 3 (15)% for the bvFTD group, and in the distractor interest area was − 10 (16)% for the control group compared with − 2 (13)% for the bvFTD group (Table 2, Fig. 2).

**Table 2:** Comparison of the average (standard deviation) mean dwell time change scores *between* control and bvFTD groups in the simple and complex emotion recognition tasks for the target, similar and distractor interest areas. Significant differences between groups are shown in bold

	Controls, average (SD)	bvFTD, average (SD)	% difference between groups (95% confidence intervals)
**Simple**			
Target	35 (27)	4 (18)	**31 (2, 38)**
Similar	**−** 10 (15)	− 3 (15)	**− 7 (− 10, − 3)**
Distractor	**−** 10 (16)	− 2 (13)	**− 8 (− 10, − 4)**
**Complex**			
Target	15 (26)	2 (18)	**− 13 (8, 19)**
Similar	**−** 2 (19)	**−** 1 (15)	1 (**−** 4, 3)
Distractor	**−** 2 (18)	**−** 3 (14)	1 (**−** 4, 1)

Within the control group, there was a significant difference in the mean dwell time change scores between the target and similar interest areas (45%), and target and distractor interest areas (44%), but not the similar and distractor interest areas (0%) (Table 3, Fig. 2). Within the bvFTD group, there was a similar pattern but to a lesser extent: there was a significant difference in the mean dwell time change scores between the target and similar interest areas (7%), and target and distractor interest areas (6%), but not the similar and distractor interest areas (− 1%) (Table 3, Fig. 2).

**Table 3:** Comparison *within* each of the control and bvFTD groups of the average mean dwell time change scores across interest areas (target vs. similar, target vs. distractor, and similar vs. distractor) in the simple and complex emotion recognition tasks. Significant differences between groups are shown in bold

			% difference between interest areas (95% confidence intervals)
**Simple**	Controls	Target vs similar	**45 (36, 52)**
		Target vs distractor	**44 (35, 52)**
		Similar vs distractor	0 (− 1, 1)
	bvFTD	Target vs similar	**7 (4, 14)**
		Target vs distractor	**6 (3, 14)**
		Similar vs distractor	− 1 (− 3, − 1)
**Complex**	Controls	Target vs similar	**17 (13, 22)**
		Target vs distractor	**18 (14, 23)**
		Similar vs distractor	0 (− 2, 2)
	bvFTD	Target vs similar	**4 (0, 9)**
		Target vs distractor	**5 (2, 10)**
		Similar vs distractor	1 (−1, 3)

A similar pattern of results was seen in the complex emotion recognition task, with the control group spending significantly more time looking at the target image after the emotion word was presented than the bvFTD group (*p* < 0.05): the average (standard deviation) mean dwell time change score in the target interest area was 15 (26)% for the control group compared with only 2 (18)% for the bvFTD group (Table 2, Fig. 2). However, there was no difference between the groups in the similar or distractor interest areas.

Also similarly to the simple emotion recognition task, there was a significant difference in the mean dwell time change scores between the target and similar interest areas (17%), and target and distractor interest areas (18%), but not the similar and distractor interest areas (0%) in the control group for the complex emotion recognition task (Table 3, Fig. 2). In the bvFTD group, these differences were also significant between the target and similar interest areas (4%), and target and distractor interest areas (5%), but not the similar and distractor interest areas (1%) (Table 3, Fig. 2).

**Fig. 4:**
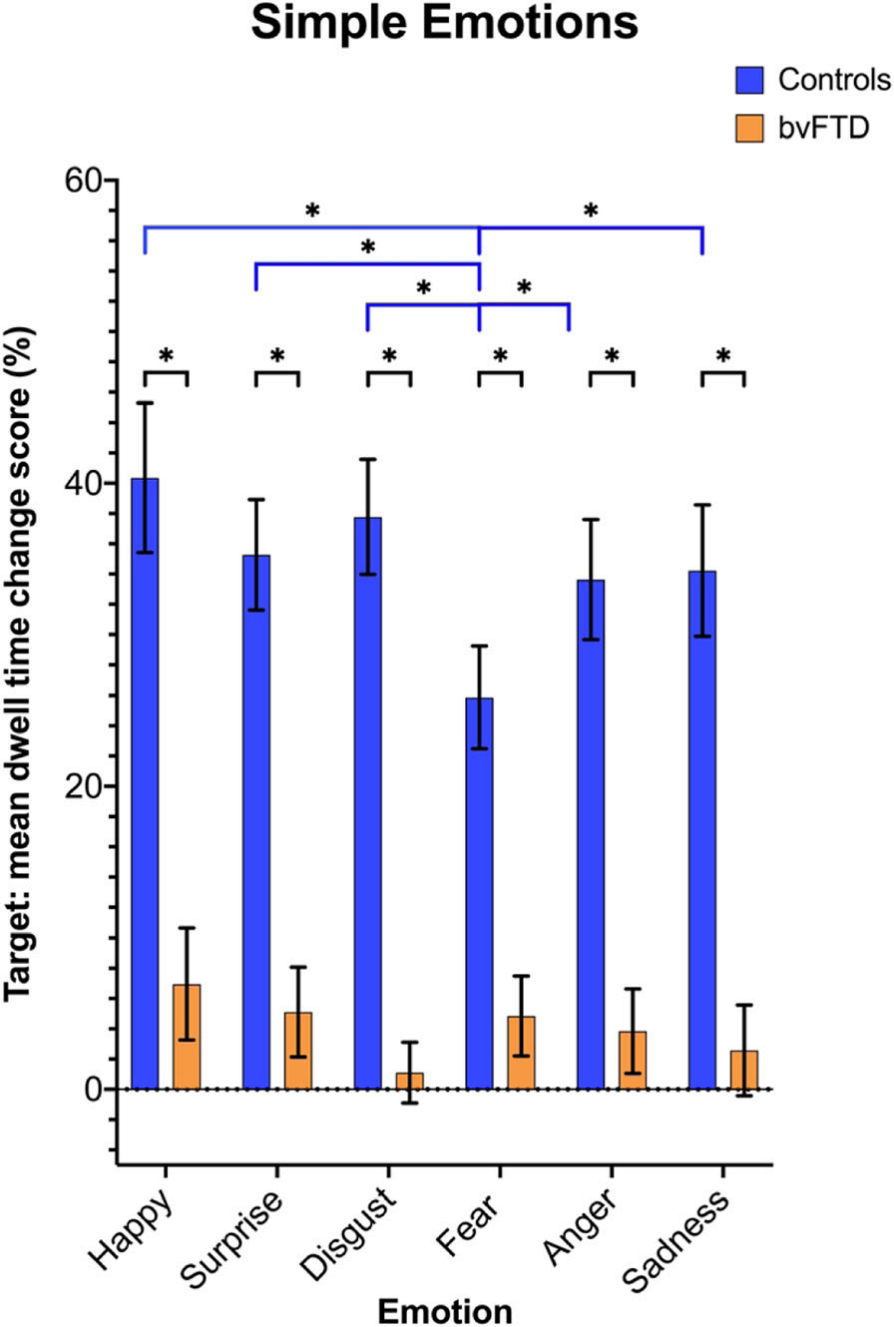
Mean dwell time change scores for bvFTD and control groups for the target interest area in the individual emotions in the simple emotion recognition task. Blue significance lines represent within the control group differences across the emotions, whilst the black significance lines represent significant differences between the control and bvFTD groups on each emotion

The bvFTD group had a significantly lower average mean dwell time change score on all six basic emotions than the control group in the simple emotion recognition task (Fig. 4, Supplementary Table 2). Within the control group, the performance was similar across all emotions, except for fear where the average mean dwell time change score was significantly less than all of the other emotions (Fig. 4, Supplementary Table 3). No significant differences were seen in the bvFTD group across the emotions (Fig. 4, Supplementary Table 3).

The mean dwell time change score for the target interest area in the bvFTD group significantly (negatively) correlated with performance on the D-KEFS Color-Word Inteference test (rho = − 0.42, *p* = 0.042) for the complex emotion recognition task (Supplementary Table 4), but not for the simple emotion recognition task (rho = − 0.34, *p* = 0.128). Although the rho was similar for the correlations of the eye tracking tasks with the social cognition test used (mini-SEA), neither was significant (for simple emotion recognition task, rho = 0.38, *p* = 0.178; for complex emotion recognition task, rho = 0.36, *p* = 0.195). There were no other significant correlations with the neuropsychological tasks including with the language task (Supplementary Table 4).

The mean dwell time change score for the target interest area in the bvFTD group significantly (positively) correlated with the volume of the right ventromedial prefrontal cortex (rho = 0.33, *p* = 0.022) and right orbitofrontal cortex (rho = 0.33, *p* = 0.031) in the complex emotion recognition task (Table 4), and although there were no significant correlations in the simple emotion recognition task, the rho value was highest in the same regions of interest: the right ventromedial prefrontal cortex (rho = 0.26, *p* = 0.079) and right orbitofrontal cortex (rho = 0.26, *p* = 0.107).

**Table 4:** Correlations between the mean dwell time change scores for the target interest area and the neuroanatomical regional volumes within the bvFTD group in the simple and complex emotion recognition tasks. Bold indicates a significant correlation

	Simple		Complex	
	Rho	*p*	Rho	*p*
Orbitofrontal cortex				
Left	0.18	0.303	0.21	0.176
Right	0.26	0.107	**0.33**	**0.031**
Dorsolateral prefrontal cortex				
Left	0.07	0.762	0.12	0.561
Right	0.10	0.615	0.17	0.396
Ventromedial prefrontal cortex				
Left	0.11	0.594	0.12	0.534
Right	0.26	0.079	**0.33**	**0.022**
Temporal cortex				
Left	− 0.11	0.656	− 0.08	0.731
Right	0.19	0.394	0.20	0.356
Parietal cortex				
Left	− 0.34	0.144	− 0.18	0.500
Right	− 0.05	0.855	0.14	0.577
Occipital cortex				
Left	− 0.44	0.107	− 0.25	0.420
Right	− 0.25	0.351	− 0.12	0.656
Striatum				
Left	− 0.03	0.919	0.00	0.989
Right	0.08	0.776	0.14	0.600
Amygdala				
Left	0.08	0.711	0.08	0.729
Right	0.22	0.300	0.20	0.333

## Discussion

In this study, we show that instructionless eye tracking tasks are able to detect simple and complex emotion recognition deficits in individuals with bvFTD and that lower mean dwell time change scores correlate with atrophy of the right orbitofrontal and ventromedial prefrontal cortex.

We have developed a short, simple, test of social cognition with essentially no test instructions, hence reducing difficulties that occur in more difficult tasks due to impaired comprehension. Importantly, controls do not score at a ceiling level, unlike many standard social cognition tasks, and furthermore, all of the individuals with bvFTD, who in this study were mildly to moderately impaired, were able to complete the tests. Future studies examining whether there are practice effects and the validity of the tasks over time will be important.

In both tasks, as hypothesised, the control group had a positive dwell time change score for the target interest area (35% for the simple and 15% for the complex task) and a negative dwell time change score for the non-target interest areas (− 10 for the simple and − 2 for the complex task). We predicted that the bvFTD group would have a dwell time change score approaching zero for both target and non-target interest areas. Instead, as a group, the dwell time change score for the target interest area was 4% for the simple task and 2% for the complex task, and for the non-target interest areas was − 1 to − 3%. Whilst significantly lower than the control group, it can be seen from Fig. 2 that there are a small number of individuals who seem to be able to perform the task well, their score overlapping with that of the control group. Further work in larger groups, and on a longitudinal basis, will be needed to study such participants, in order to understand differential performance and its underlying pathophysiology.

A larger overlap in mean dwell time change scores for bvFTD compared with controls was seen in the complex emotion recognition task compared with the simple task. Whilst this suggests the simple emotion recognition tasks may be more helpful in this bvFTD population in diagnosing social cognitive impairment, this may not be the case for those with either very early bvFTD or who are presymptomatic (i.e. those who are in the prodromal stage of genetic FTD). The increased difficulty of the complex task means that it may potentially be more sensitive to subtle changes at this stage of the disease when performance may remain normal on the simple emotion recognition task. Investigation of presymptomatic genetic FTD mutation carriers will be helpful to understand this better, and particularly studying people longitudinally as they phenoconvert.

When looking at performance across the individual emotions in the simple emotion recognition task, the control group had a decrease in their ability to identify fearful expressions when compared to the other emotions. This is consistent with prior literature showing fear is one of the most difficult of the basic emotions to recognise [28]. However, there were no significant differences observed between the emotions in the bvFTD group, which is different than a number of other prior studies in FTD which show worse performance on negative emotions compared with positive emotions [6–8].

The only significant correlation seen with standard ‘pen and paper’ cognitive tests was the complex emotion recognition task with the D-KEFS Color-Word Interference Test, although even this was a relatively weak correlation. There was a similar trend with the simple emotion recognition task, suggesting that both tests may have an executive function component to them. In contrast, although the rho value was similar, we did not find evidence of a significant correlation with scores on the mini-SEA (or its individual subtests), the standard social cognition test performed in all of the participants. The association with executive function but not social cognition may be due to a number of reasons: firstly, the tasks may assess more subtle deficits than picked up through the standard social cognition test (as they were in fact designed to do, and was seen in a previous novel eye tracking test that was able to identify more individuals as having deficits than the standard pen and paper task [22]); secondly, there is a close interrelationship between executive function and many aspects of social cognition as highlighted by previous studies [29–31]; thirdly, the small sample size may not be able to pick up a significant correlation with the mini-SEA (as nonetheless the rho value was 0.38 for the simple task and 0.36 for the complex task); lastly, and as with many psychometric tests, the tasks may well tap into multiple cognitive components in brain function even if primarily a social cognition task.

The neuroimaging analysis demonstrated an association of lower mean dwell time change score with atrophy of the right ventromedial prefrontal cortex. This is consistent with previous findings that the right ventromedial prefrontal cortex plays a central role in social cognition and the recognition of emotions in faces [32, 33]. An association was also seen with the right orbitofrontal cortex, an area involved in social decision-making [34], and previously identified as linked to emotion recognition deficits in individuals with bvFTD [11, 35]. Whilst these findings provide some support that the novel eye tracking tasks may be measuring social cognition, these regions are also implicated in other cognitive domains.

Overall, we have developed a novel set of tasks which allow detection of impaired social cognition in FTD. The study adds to the literature showing the presence of emotion recognition deficits in FTD but the nature of these novel tasks means that more subtle deficits may be detectable compared to prior tests. Further studies in presymptomatic genetic FTD populations such as the GENFI (www.genfi.org) or ALLFTD (www.allftd.org) studies will be important to see how early social cognition difficulties can be seen in the disease process. This has implications for future clinical trials in terms of stratifying participants, but also in detecting deficits that might help make earlier diagnoses of bvFTD. We believe that this initial exploratory study also provides the theoretical basis for developing further instructionless eye tracking tasks that could detect other subdomains of social cognition impairment such as theory of mind and moral reasoning.

### Limitations

The study has a number of limitations. Firstly, whilst the sample size used in this study is typical of those investigating bvFTD, given the rarity of the condition, the study would benefit from a replication in a larger cohort. Secondly, as with all neuropsychometric tests, it is difficult to assess whether or not a task is assessing a specific cognitive domain or whether other abilities are influencing one’s performance on a task, for example executive function having an impact on social cognitive abilities as mentioned above. The tasks in this study have been developed to remove as many confounding factors as possible by keeping them simple and instructionless, but further studies in other disorders that have impaired social cognition but intact executive function and other cognitive domains will be helpful to understand the task further. Thirdly, it is possible that the individuals with bvFTD, who were impaired on a language task compared with controls, are having trouble comprehending the emotional words for the complex task which may be limiting their ability to do the eye tracking tests. However, there was no correlation of scores on either of the tests with the language task. Fourthly, there was no significant difference in scores for the ‘similar’ and ‘distractor’ items on either the simple or complex tasks. This suggests that both are acting just as non-target items and future analyses should focus on ‘target’ and ‘non-target’ interest areas only. Lastly, a better understanding of longitudinal performance and the effects of repeated testing is needed, although the correct answers are never given to participants, potentially limiting any practice effects.

## Conclusions

In summary, the results suggest that instructionless eye tracking tests are a viable tool for assessing social cognition in bvFTD. Further work in a larger control population and other disorders with social cognition deficits will be needed to better understand the replicability and reliability of the task but these novel tasks open the opportunity for a quantitative measure of social cognition that may well be helpful as outcome measures in future trials.

## Supplementary Information

**Additional file 1.**

## Data Availability

The datasets generated during and/or analysed during the current study are not publicly available, as the conditions of our ethical approval do not permit public archiving of individual anonymised data, but are available from the corresponding author on reasonable request.
