# Supplementary material for: Novel instructionless eye tracking tasks identify emotion recognition deficits in frontotemporal dementia
Source: Alzheimers Res Ther. 2021 Feb 8;13:39. doi: 10.1186/s13195-021-00775-x (PMC7871635; doi:10.1186/s13195-021-00775-x)
Supplement: Supplementary file 1 — Additional file 1. [file 13195_2021_775_MOESM1_ESM.docx]

**Supplementary Data**

Table S1: Mean and standard deviation (SD) for the amplitude error, saccade latency and peak velocity (vertical and horizontal) during the pro-saccade task in the controls and bvFTD group.

|  | Direction | Visual angle | Controls | bvFTD | p value |
| --- | --- | --- | --- | --- | --- |
|  |  |  | Mean (SD) | Mean (SD) |  |
| Amplitude error  (degrees of visual angle) | Horizontal | 8 | -4.7 (0.5) | -4.9 (0.7) | 0.408 |
|  | Vertical | 5 | -2.6 (0.4) | -2.7 (0.4) | 0.534 |
| Saccade latency  (milliseconds) | Horizontal | 8 | 201.6 (49.2) | 249.6 (196.2) | 0.191 |
|  | Vertical | 5 | 255.6 (59.5) | 312.8 (334.7) | 0.487 |
| Peak velocity  (degrees per second) | Horizontal | 8 | 199.4 (29.4) | 207.0 (40.7) | 0.200 |
|  | Vertical | 5 | 150.8 (32.4) | 165.5 (34.1) | 0.124 |

Table S2: Comparison of the average (standard deviation) mean dwell time change scores between control and bvFTD groups in the individual emotions in the simple emotion processing task for the target interest area. Significant differences between groups are shown in bold.

|  | Control  Average (SD) | bvFTD  Average (SD) | % difference between groups  (95% confidence intervals) |
| --- | --- | --- | --- |
| Happy | **40 (29)** | **7 (20)** | **-33 (-48, -24)** |
| Surprise | **35 (25)** | **5 (19)** | **-30 (-43, -23)** |
| Disgust | **37 (27)** | **1 (16)** | **-36 (-49, -29)** |
| Fear | **26 (29)** | **4 (19)** | **-22 (-34, -14)** |
| Anger | **35 (26)** | **5 (16)** | **-30 (-31, -22)** |
| Sadness | **34 (25)** | **3 (18)** | **-31 (-46, -23)** |
| *Positive emotions combined* | ***38 (27)*** | ***6 (3)*** | ***-32 (-42, -22)*** |
| *Negative emotions combined* | ***33 (27)*** | ***3 (18)*** | ***-30 (-38, -22)*** |

Table S3: Comparison within each of the control and bvFTD groups of the average mean dwell time change scores within the target interest area across the individual emotions in the simple emotion processing task. Mean differences and p values are shown with significant differences between emotions shown in bold.

|  | | Anger | | Disgust | | Fear | | Happy | | Sadness | | Surprise | |
| --- | --- | --- | --- | --- | --- | --- | --- | --- | --- | --- | --- | --- | --- |
| Controls | Anger |  |  | 4% | 0.388 | **-8%** | **0.014** | 6% | 0.066 | 0% | 0.940 | 0% | 0.657 |
|  | Disgust |  |  |  |  | **-12%** | **<0.001** | 3% | 0.471 | -4% | 0.352 | -3% | 0.432 |
|  | Fear |  |  |  |  |  |  | **15%** | **<0.001** | **8%** | **0.024** | **10%** | **0.001** |
|  | Happy |  |  |  |  |  |  |  |  | -6% | 0.072 | -5% | 0.980 |
|  | Sadness |  |  |  |  |  |  |  |  |  |  | 0% | 0.755 |
|  | Surprise |  |  |  |  |  |  |  |  |  |  |  |  |
| bvFTD | Anger |  |  | -4% | 0.163 | -1% | 0.479 | 2% | 0.548 | -2% | 0.183 | 0% | 0.972 |
|  | Disgust |  |  |  |  | 3% | 0.246 | 6% | 0.057 | 2% | 0.477 | 4% | 0.152 |
|  | Fear |  |  |  |  |  |  | 3% | 0.371 | -1% | 0.477 | 1% | 0.561 |
|  | Happy |  |  |  |  |  |  |  |  | -4% | 0.098 | -2% | 0.518 |
|  | Sadness |  |  |  |  |  |  |  |  |  |  | 3% | 0.232 |
|  | Surprise |  |  |  |  |  |  |  |  |  |  |  |  |

**Table S4: Correlations between the mean dwell time change scores for the target interest area and cognitive task scores within the bvFTD group in the simple and complex emotion processing tasks.** **Bold indicates a significant correlation.**

|  | Simple | | Complex | |
| --- | --- | --- | --- | --- |
|  | rho | p | rho | p |
| WMS-R Digit span forwards | 0.16 | 0.604 | 0.26 | 0.330 |
| WMS-R Digit span backwards | 0.33 | 0.384 | 0.31 | 0.360 |
| Phonemic fluency | 0.09 | 0.771 | 0.40 | 0.886 |
| D-KEFS Color-Word Interference Test | -0.34 | 0.128 | **-0.42** | **0.042** |
| Trail Making Test part A | -0.37 | 0.077 | -0.29 | 0.184 |
| Trail Making Test part B | -0.21 | 0.528 | -0.35 | 0.197 |
| Graded Naming Test | 0.23 | 0.247 | 0.20 | 0.376 |
| Mini-Social and Emotional Assessment total | 0.38 | 0.178 | 0.36 | 0.195 |
| Mini-Social and Emotional Assessment Faux-Pas subtest | 0.41 | 0.218 | 0.27 | 0.458 |
| Mini-Social and Emotional Assessment Emotion Recognition subtest | 0.15 | 0.485 | 0.25 | 0.213 |
